# Single step synthesis of highly conductive room-temperature stable cation-substituted mayenite electride target and thin film

**DOI:** 10.1038/s41598-019-41512-7

**Published:** 2019-03-21

**Authors:** Karim Khan, Ayesha khan Tareen, Usman Khan, Adeela Nairan, Sayed Elshahat, Naseer Muhammad, Muhammad Saeed, Ashish Yadav, Luigi Bibbò, Zhengbiao Ouyang

**Affiliations:** 1 College of Electronic Science and Technology of Shenzhen University, THz Technical Research Center of Shenzhen University, Key Laboratory of Optoelectronics Devices and Systems of Ministry of Education and Guangdong Province Shenzhen University, Shenzhen, 518060 P. R. China; 2 Ningbo Institute of Material Technology and Engineering, Chinese Academy of Sciences, Ningbo, 315201 P. R. China; 3 Low dimensional materials and devices laboratory, Tsinghua-Berkeley Shenzhen institute, Tsinghua University Shenzhen, Shenzhen, 518055 P. R. China; 4 Division of Energy and Environment, Graduate School at Shenzhen, Tsinghua University, Shenzhen, 518055 P. R. China; 5 Institute for Advanced Study, Shenzhen University, Shenzhen, Guangdong, 518060 P. R. China

## Abstract

Novel approaches to synthesize efficient inorganic electride [Ca_24_Al_28_O_64_]^4+^(e^−^)_4_ (thereafter, C12A7:e^−^) at ambient pressure under nitrogen atmosphere, are actively sought out to reduce the cost of massive formation of nanosized powder as well as compact large size target production. It led to a new era in low cost industrial applications of this abundant material as Transparent Conducting Oxides (TCOs) and as a catalyst. Therefore, the present study about C12A7:e^−^ electride is directed towards challenges of cation doping in C12A7:e^−^ to enhance the conductivity and form target to deposit thin film. Our investigation for cation doping on structural and electrical properties of Sn- and Si-doped C12A7:e^−^ (Si-C12A7:e, and Sn-C12A7:e^−^) reduced graphene oxide (rGO) composite shows the maximum achieved conductivities of 5.79 S·cm^−1^ and 1.75 S·cm^−1^ respectively. On the other hand when both samples melted, then rGO free Sn-C12A7:e^−^ and Si-C12A7:e^−^ were obtained, with conductivities ~280 S.cm^−1^ and 300 S·cm^−1^, respectively. Iodometry based measured electron concentration of rGO free Sn-C12A7:e^−^ and Si-C12A7:e^−^, 3 inch electride targets were ~2.22 × 10^21^ cm^−3^, with relative 97 ± 0.5% density, and ~2.23 × 10^21^ cm^−3^ with relative 99 ± 0.5% density, respectively. Theoretical conductivity was already reported excluding any associated experimental support. Hence the above results manifested feasibility of this sol-gel method for different elements doping to further boost up the electrical properties.

## Introduction

Electrides are exotic ionic-solid materials in solid state with cavity-trapped electrons occupies crystallographic site that serve as smallest possible anions^1–9^. The cavity-trapped electrons are neither localized on specific atoms/molecules, nor delocalized as in metals, but rather occupy sites of anions (Cl^−^, OH^−^ etc.)^6^. Two major classes of electride, organic and inorganic, have been developed so far^4,6,10,11^. Most organic electrides are, unfortunately, unstable at room temperature, and get degrade after exposure to air or moisture. The first inorganic high temperature stable electride in which trapped electrons counter cations occupy separate sites is C12A7:e^−^, named “mayenite electride”^12,13^.

Oxymayenite, [Ca_24_Al_28_O_64_]^4+^(2O^2−^) (thereafter, C12A7) is an excellent insulator, transparent oxide material (energy band gape, Eg ~7 eV) which can be converted into conducting light metal oxide, C12A7:e^−^ ^14^. Since, the first room-temperature stable electride, C12A7:e^−^ was discovered (2003), it has attracted much attention due to its unique properties. The Insulator-Metal (I-M)-conversion can get either by removal of clathrate free oxygen from the cages or by introduction of hydrogen along with illumination by UV-radiations^15^. First method based on removal of clathrate free oxygen from cages is very important to further explore to achieve highly conductivity C12A7:e^−^ ^16^. Till now different methods are reported time by time for the conversion of insulating C12A7 into different degree of conductivity and transparency by reduction of free oxygen clathrate in cages. Each method had their own advantage and limitation, so can choose particular synthesis techniques according to application requirements^16^ e.g. complete removal of free oxygen, mass production, and electron-doped area^7,12,17,18^. It was highly desired to develop a simple, less time/energy consuming, cost-effective, and easy scale-up synthesis method for large-scale cations doping.

Microstructure control doping of C12A7:e^−^ is also an important but less experimentally studied^19^. In contrast to anionic substitutions into C12A7:e^−^ less is known about the influence of cationic substitutions on the aluminum and calcium site^19^. The availability of access free cages increases the possibility of storage of further extra “extrinsic free oxygen” in available empty cages in C12A7 unit cell. So, after doping C12A7, the excess extra free oxygen anions can go into an ionic crystal of the stoichiometric compound, resulting either in equivalent number of cations being oxidized or creation of vacancies to balance charge like in case of excess oxygen in UO_2+x_^20^ and metal deficit manganous Mn_1−x_O and ferrous oxides Fe_1−x_O^19^. Hence, after reduction of doped C12A7:e^−^ further extra electron, rather than that of intrinsic electride (~2.3 × 10^23^ cm^−3^), can accommodate till theoretical value of ~6 × 10^23^ cm^−3^ and hence corresponding conductivity will further increased^19^. Huang *et al*. based on DFT simulations give a detail explanations about the role of cationic (Mg^2+^, Cu^2+^, Sr^2+^, Fe^3+^, Ir^4+^,P^5+^, and V^5+^) substitutions and also theoretically evaluate the structural and electronic features of C12A7:e^−^ ^19^. Theoretical based experimental studies has been done for some of the cation substitutions in C12A7 like, Mg, Si, Ga, Fe, V etc.^21–25^. So shortly the results of all type of doping based on previously introduced synthesis methods, except synthesis method introduced by our group, causes decrease in the electronic conductivity, except Si-substitution^21^ where conductivity increases from 0.15 to 0.61 S·cm^−1^ for x = 0 to 4 and in case of Ga-doping in C12A7 the C12A7 phase is decomposed after reduction^24^. Further improvements in synthesis techniques are required to further boost up conductivity of C12A7:e^−^ ^5,26^. New findings in the cation doped C12A7:e^−^ electride will opened up a new frontier in TCO based electronics and fuel cells^12,26–29^. The advantages of mayenite are its and its low cost precursors easy availability^16^. It is accepted that the key in the development of doped C12A7:e^−^ electride is low cost and large scale production. As conductivity of C12A7:e^−^ originate from the cage conduction bands (CCB) related to orbital of the cations in the cages framework. Therefore, cation substitutions can be expected to change the framework of nano cages, affect the CCB states, and thus alter the conductivity of the material. Therefore, the present work provides guidance and insights for exploring simple and scalable synthesis method for the novel doped C12A7:e^−^ materials with enhanced electron transport properties, via careful selection and manipulation of cations doping. Hence, we intended to apply single step facile sol-gel scheme to synthesize C12A7:e^−^ electride nano-powder doped with different suitable cations.

## Scope of Method

### Synthesis and characterization of doped C12A7:e^–^

The academic research on the C12A7:e^−^ material is constantly in progress to develop new fundamental science and their potential industrial applications. This new proposed modified sol-gel method will provide a new way for synthesis of C12A7:e^−^ with various cations doping. Enhancement in conductivity was observed experimentally for doped samples similarly reported based on theoretical calculations^30^. Flexibility associated with this synthesizing approach for synthesis of unique cage structure single phase doped C12A7:e^−^ should enable many applications and also presents opportunity for studies of suitable elements doping in C12A7:e^−^ with aim of optimizing its opto-electrical properties.

### Experimental scheme and synthesis

**Figure 1:**
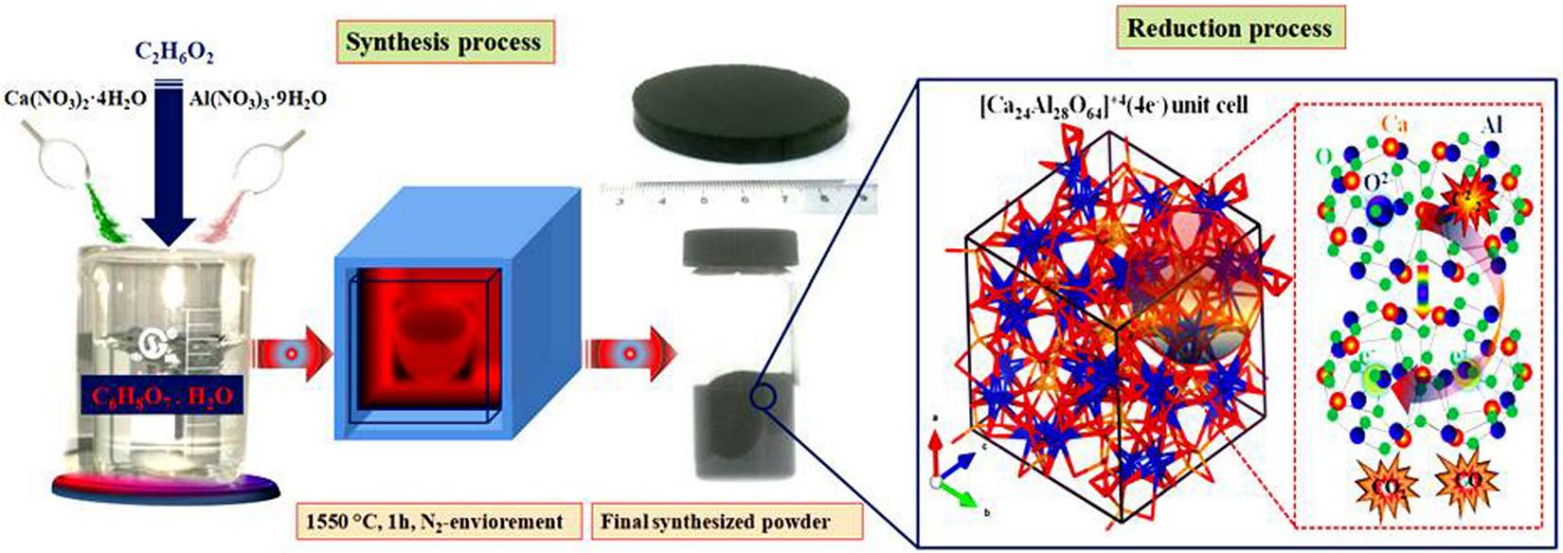
Scheme for synthesis of cation doped mayenite and its reduction.

Cation doped C12A7:e^−^ was prepared by using low cost precursors (Ca(NO_3_)_2_.4H_2_O and Al(NO_3_)_3_.9H_2_O) and using ethylene glycol (EG) as a solvent by using modified sol-gel method. First of all, stoichiometric ratios of nitrides were weighted (12:14). The raw materials for “Sn”, and “Si” doping elements used were, SnCl_4_·5H_2_O, and SiO_2_ respectively. The sources of doped elements were added to EG solution separately, along with nitrates at 60 °C under stirring condition to get transparent solution. This gel was kept at about 100 °C for one hour to vaporize physically absorbed water and then finally dried at 275 °C for 4 hours in a drier to vaporize extra EG to get dried gel. Before further heat treatment, the resulting dried gel was crushed into powder and was further heat treated at 500 °C for 1 h in a nitrogen environment with 5 °C/min increase rate. The product was again crushed into powder and then divided into two parts, one was pressed in pellet shape at 150 MPa and the other was directly used to get a conductive powder. Finally, the resultant powder and pellets were sintered in alumina crucible under nitrogen atmosphere with a heating rate of 4 °C/min and kept at 1550 °C for 1 h (Fig. 1). Finally we melted samples at 1700 °C for 30 min under nitrogen environment, and hence solid state migration of carbon species outside of mayenite structure occurs, hence after polishing the melted target we can got rGO free doped C12A7:e^−^ target to deposit thin film.

## Results and Discussion

X-ray powder diffraction (XRD) analysis was performed to study the obtained phase microstructures. The Raman spectra were excited by a 532 nm air-cooled argon ion laser (20 mW) to characterization/investigate the molecule structure and bonding of the mayenite electride and graphitic materials. Electrical conductivity was measured by four probe method, where Pt-past was used to further improve the contact. The simple and accurate iodometry technique was used to determine electrons concentration^9^. Scanning electron microscope (SEM) and Transmission Electron Microscopy (TEM) were used to study the microstructure and morphology of final products. The X-ray photoelectron spectroscopy (XPS) was used for elemental analysis along with bonding formations between those elements to verify required final material synthesis. Now we are going to discuss each characterization method results in detail^1,2^.

### Sn-doped C12A7:e^−^

#### Crystalline phase analysis

**Figure 2:**
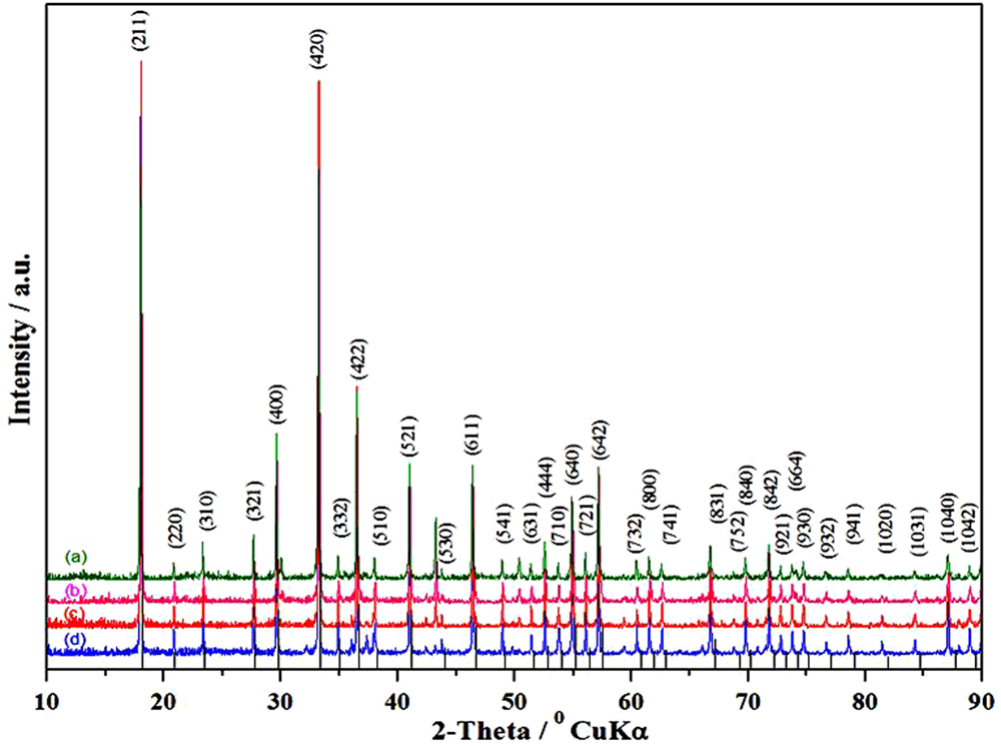
XRD patterns of Sn-doped composite, where doping level, x = (**a**) 1, (**b**) 0.75, (**c**) 0.50, (**d**) 0.25, heated at 1550 °C. For comparison, the lines on the x-axis correspond to the peak positions for the pure C12A7.

In this section first of all we studied the single step based synthesis of Sn-doped C12A7:e^−^. The XRD based study of Sn-doped C12A7:e^−^ regarding to the phase identification and crystallinity were observed with all doping levels shown in Fig. 2. All peaks are corresponding to the well crystalline C12A7 phase (JCPDS, CAS number 48–1882)^1,2,8,9^.

Doping didn’t change the basic structure of the lattice framework of C12A7 under high temperature treatment and no secondary impurity phase(s) was (were) observed due to decomposition of mayenite electride. That is because of the presence of carbon species which are in form of like rGO with slightly other graphitic materials properties, produced by thermal reduction of ethylene glycol. This acts as a template, which also makes this material stable at this high temperature^1,2,8,9^.

### Microstructural analysis

**Figure 3:**
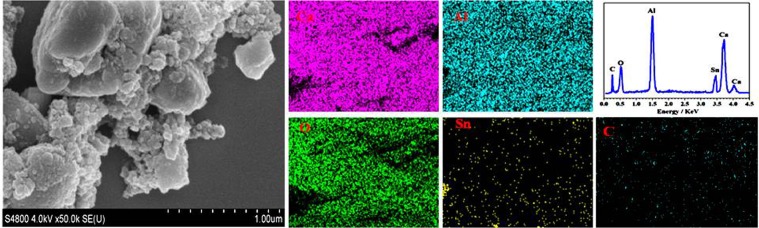
SEM images and EDX mapping of Sn-doped C12A7:e^−^ composite sample synthesis at 1550 °C, where x = 1.

The SEM images of Sn-doped C12A7:e^−^ (C_12_A_7-x_Sn_x_:e^−^, where x = 0 to 1) samples synthesized after annealing at 1550 °C are shown in Fig. 3. The Sn-doped C12A7:e^−^ sample tended to form nano size particles. In Fig. 3, EDS mapping of Sn-doped C12A7:e^−^ shows all expected elements, Ca, Al, O, C and Sn in synthesized Sn-doped C12A7:e^−^ sample^1,2,8,9^. Now for further application point of view we are going to discuss the measured electrical properties of synthesized material.

### Electrical properties study

**Figure 4:**
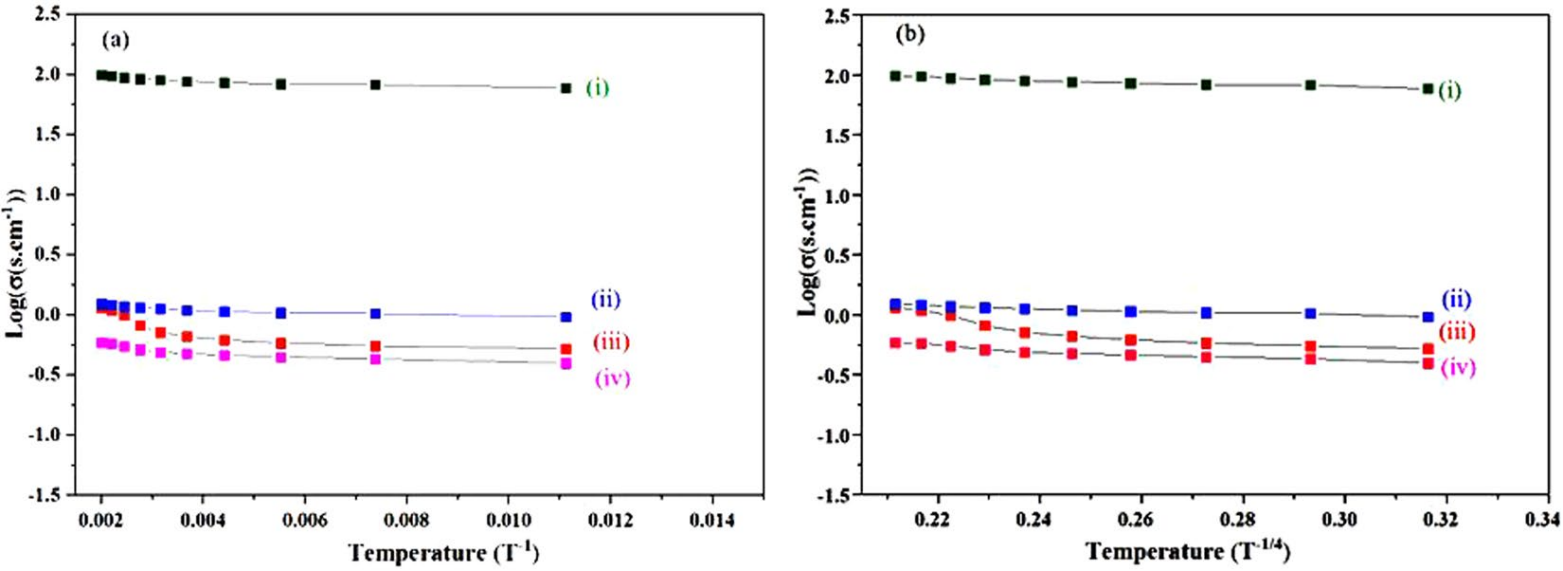
Log(σ) Vs (T^−1^, T^−1/4^ (K)) of Sn-doped composite, with doping levels of x = , (I) 1, (ii) 0.75, (iii) 0.50, and (iv) 0.25.

Another important characterization is the measuring of conductivity (Fig. 4). In case of Sn-doped C12A7:e^−^ electrical conductivity was in the range of 1.6 S·cm^−1^ to 5.79 S·cm^−1^.

The rGO free Sn-doped C12A7:e^−^ sample melted at 1700 °C for 30 min under N_2_ with highest conductivity of 280 S.cm^−1^ and Iodometry based measured electron concentration was ~2.22 × 10^21^ cm^−3^ ^1,2^ Hence we first time synthesized a 3 inch size electride target with relative density of 97 ± 0.5%, and it, will bring great revolution in opto-electronic industry. Hence, in this experiment, we successfully synthesized the Sn-C12A7:e^−^ free of rGO as a pure phase and showed its increase in conductivity behavior with doping level and its stability on reduction^31^. Therefore we also check this synthesis method for other element to check its universality for other elements doping in C12A7:e^−^.

### Si-doped C12A7:e^−^

**Figure 5:**
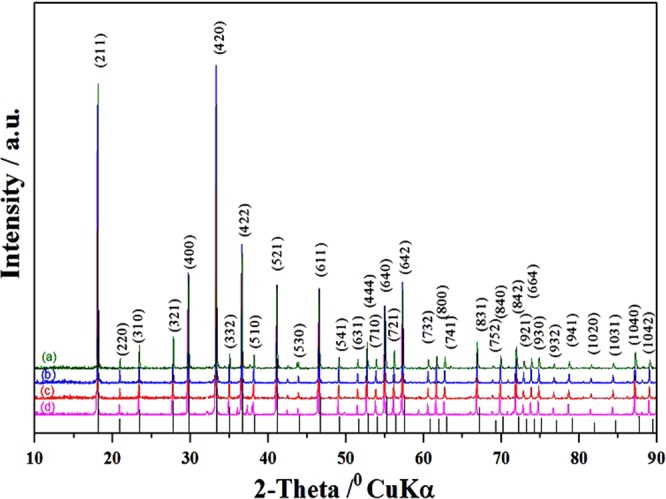
XRD patterns of Si-doped C12A7:e composite, where x = (**a**) 1, (**b**) 0.75, (**c**) 0.50, (**d**) 0.25. For comparison, lines on x-axis correspond to peak positions for the pure C12A7.

The heat treatment at 1550 °C/1 h for all samples doped with different Si values, i.e., x = 0.25, 0.50, 0.75, and 1, under N_2_ gas atmosphere for 1 h were carried out. The XRD patterns of all the Si-doped samples are shown in Fig. 5. All the peaks corresponds to the well crystalline C12A7 phase (JCPDS, CAS number 48–1882) without any second impurity phase peak due to decomposition of C12A7 phase.

The reduction treatment did not change the basic structure of the lattice framework of C12A7 under high temperature treatment and with increased doping level.

**Figure 6:**
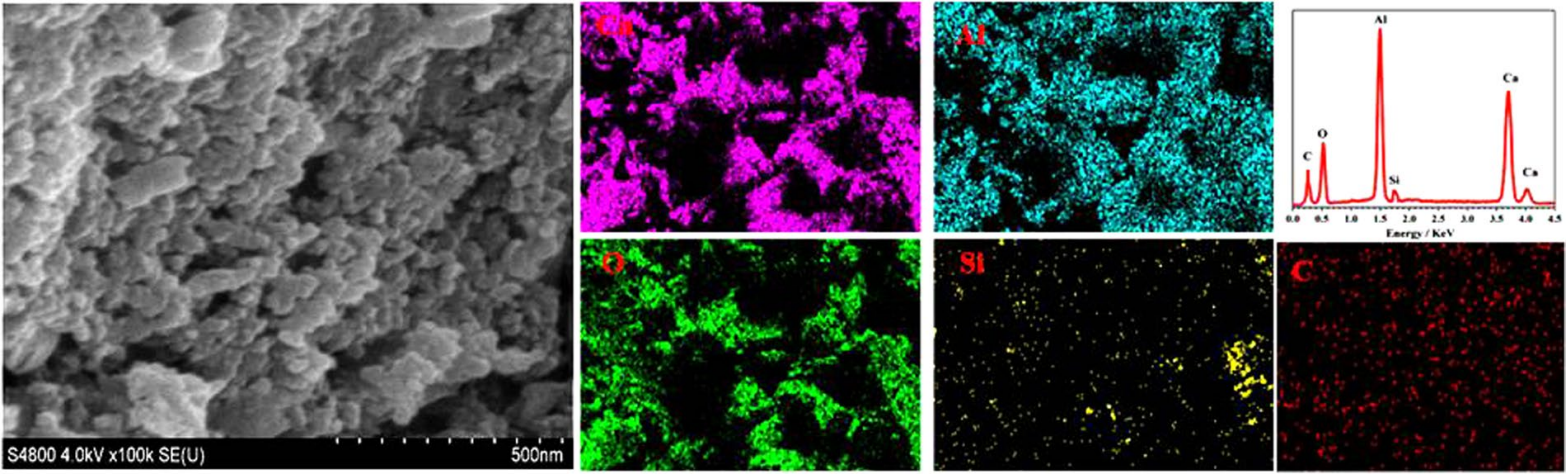
SEM images and EDX based mapping of Si-doped C12A7:e^−^ composite sample synthesis at 1550 °C where x = 1.

The surface morphological study of Si-doped C12A7:e^−^ showed nano sized particles. Such porous type nano size particles may further enhance the specific surface area and could be isolated completely by dissolving in some solvent followed by ultra sonication. Figure 6 shows all expected elements, Ca, Al, O C, and Sn in Si-doped C12A7:e^−^ sample. For further particles size analysis we studied TEM of synthesized powder.

**Figure 7:**
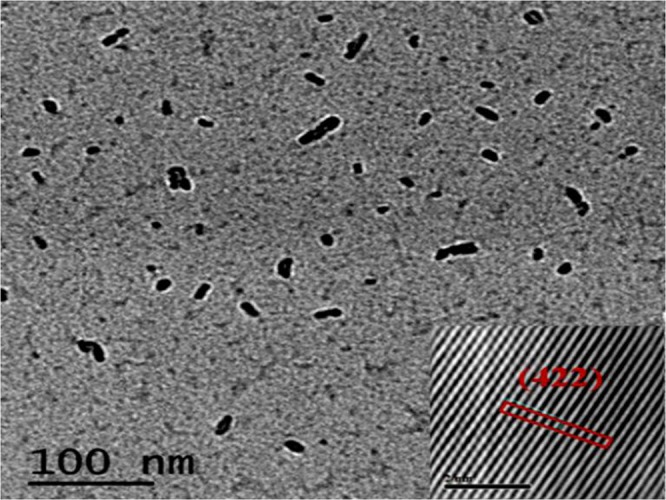
TEM images of Si-doped C12A7:e^−^ composite sample synthesis at 1550 °C where x = 1.

**Figure 8:**
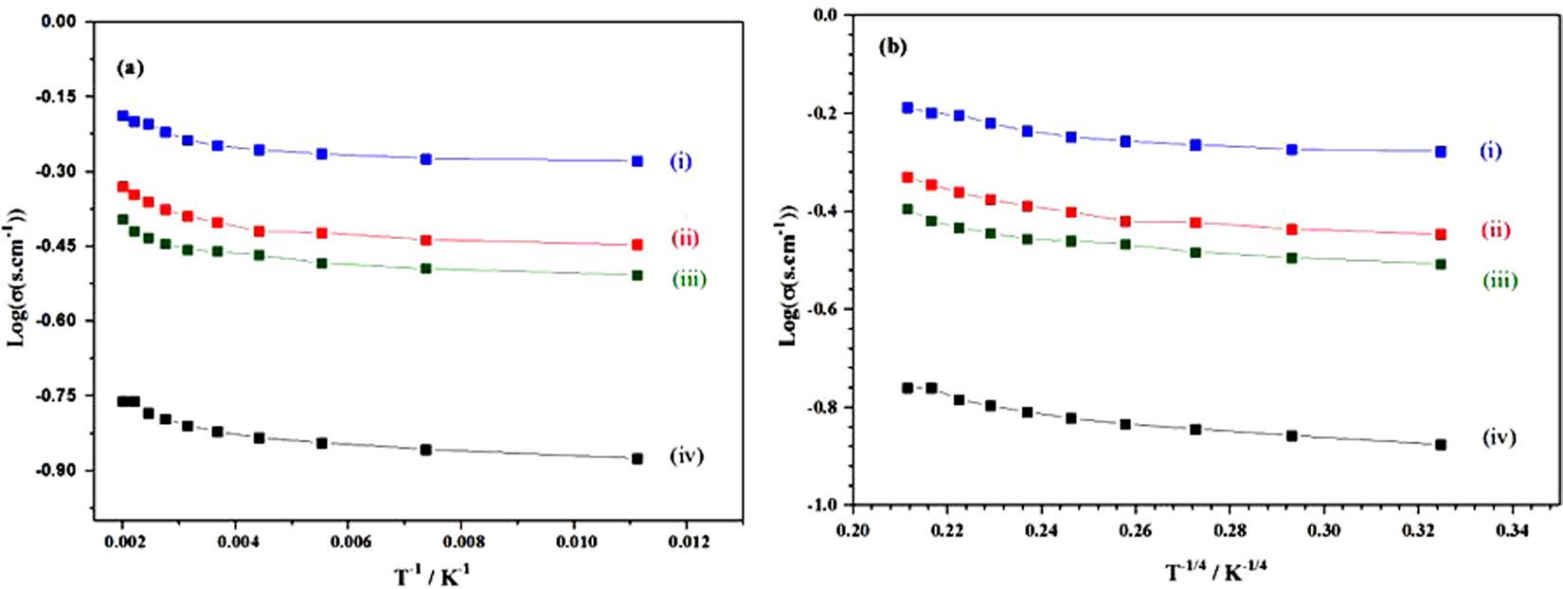
Log(σ) (σ, conductivity) Vs temperature (T^−1^, T^−1/4^ (K)) graph of C12A7:e^−^ composite samples synthesis with different Si-doping levels of x = , (i) 1, (ii) 0.75, (iii) 0. 50, and (iv) 0.25.

Figure 7 shows TEM image, where nano-size of synthesized particles were confirmed, and nano-size formation will further boost the industrial properties of this material, especially as electrocatalyst. Inset Fig. 7 shows HR-TEM image, which confirmed the C12A7:e^−^ particles, was also supported by XRD. For industrial application, important point of synthesized material is to study the conductivity (Fig. 8). In case of the Si-doped C12A7:e^−^ composite the conductivity was in range of 0.16 S·cm^−1^ to 1.75 S·cm^−1^ ^32^ but when we melted the sample at 1700 °C for 30 minutes under nitrogen environment, the highest conductivity achieved of rGO free Si-doped C12A7:e^−^ sample was ~300 S.cm^−1^, and iodometry based measured electron concentration was ~2.23 × 10^21^ cm^−3^. It is demonstrated that moderate conductivities were attributed to well crystalline morphology of Si-doped C12A7:e^−^. Hence, first time we synthesized rGO free, pure and stable phase of Si-doped C12A7:e^−^ electride target of 3 inch size, with relative density of 99 ± 0.5%. As TCOs, it will bring great revolution in field of the devices, especially in display devices.

### Electron Paramagnetic Resonance (EPR)

**Figure 9:**
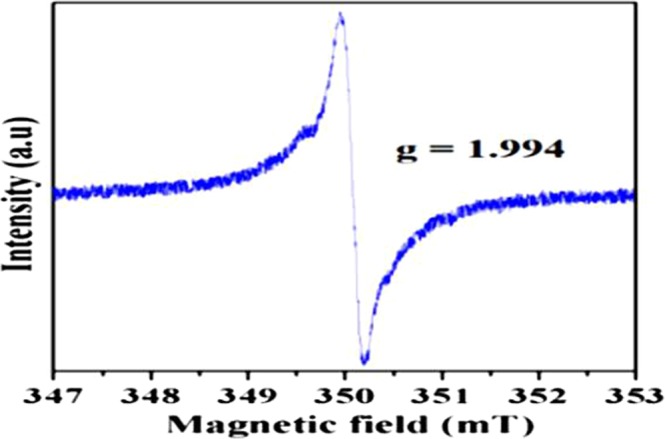
EPR spectra of carbon free Si-doped C12A7:e^−^, where x = 1.

The EPR analysis was used to calculate the density and electronic structure of trapped electrons in cages of synthesized Si-doped C12A7:e^−^ melted sample (Fig. 9). The EPR spectrum exhibits an absorption signal with at about g = 1.994, as previously reported^7^. Hence, the EPR investigation also provides the confirmation of synthesis of rGO free Si-doped C12A7:e^−^ electride.

### Raman Spectroscopy

**Figure 10:**
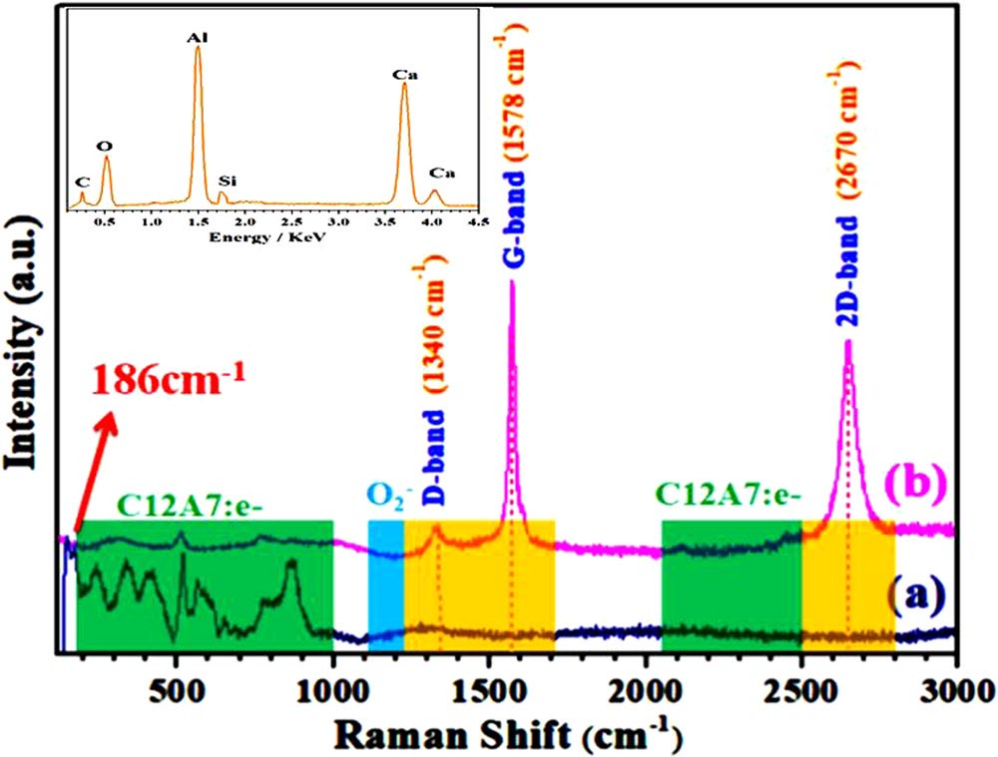
Raman spectra of synthesized Si-doped C12A7:e^−^ (**a**) melted, rGO free (**b**) composite powder with rGO.

Figure 10(b) shows Raman spectrum, where bands located at 200–1000 cm^−1^ were arose from lattice framework of C12A7^33^. The peak between 1128 to 1164 cm^−1^ are assigned as the O_2_^−^ stretching mode, were not observed, indicating the reduction of C12A7 into C12A7:e^−^^1,2^. G-band, 2D-band and D-band confirm the existence of carbon family and a very weak D band peak (1340 cm^−1^) emerges in spectra, shown defective layers^1,2,8,9^. As the peak intensity ratio I2D/IG is less than 1 and the FWHM of the 2D band peak is ~89 cm^−1^, the obtained rGO should be the stacking of multilayer sheets^1,2^. Comparatively small D band peak than 2D band peak in the Raman spectrum could be ascribed to the excellent reduction of rGO during applied synthesis process, in which the oxygen moieties were removed and the sp^2^ network was restored due to structural relaxation^2,8,9,34^. On the other hand, Fig. 10(a) shows rGO free pure Si-doped C12A7:e^−^ because the D, G, and 2D bands diapered on melting. Previous study suggested the band appear at 186 cm^−1^ is related to the electrons present in cages and its intensity is proportional to “Ne”^35^.

Inset Fig. 10 shows EDX of the melted rGO free Si-doped C12A7:e^−^, also confirmed Raman spectroscopy results.

### X-ray photoelectron spectroscopy (XPS)

**Figure 11:**
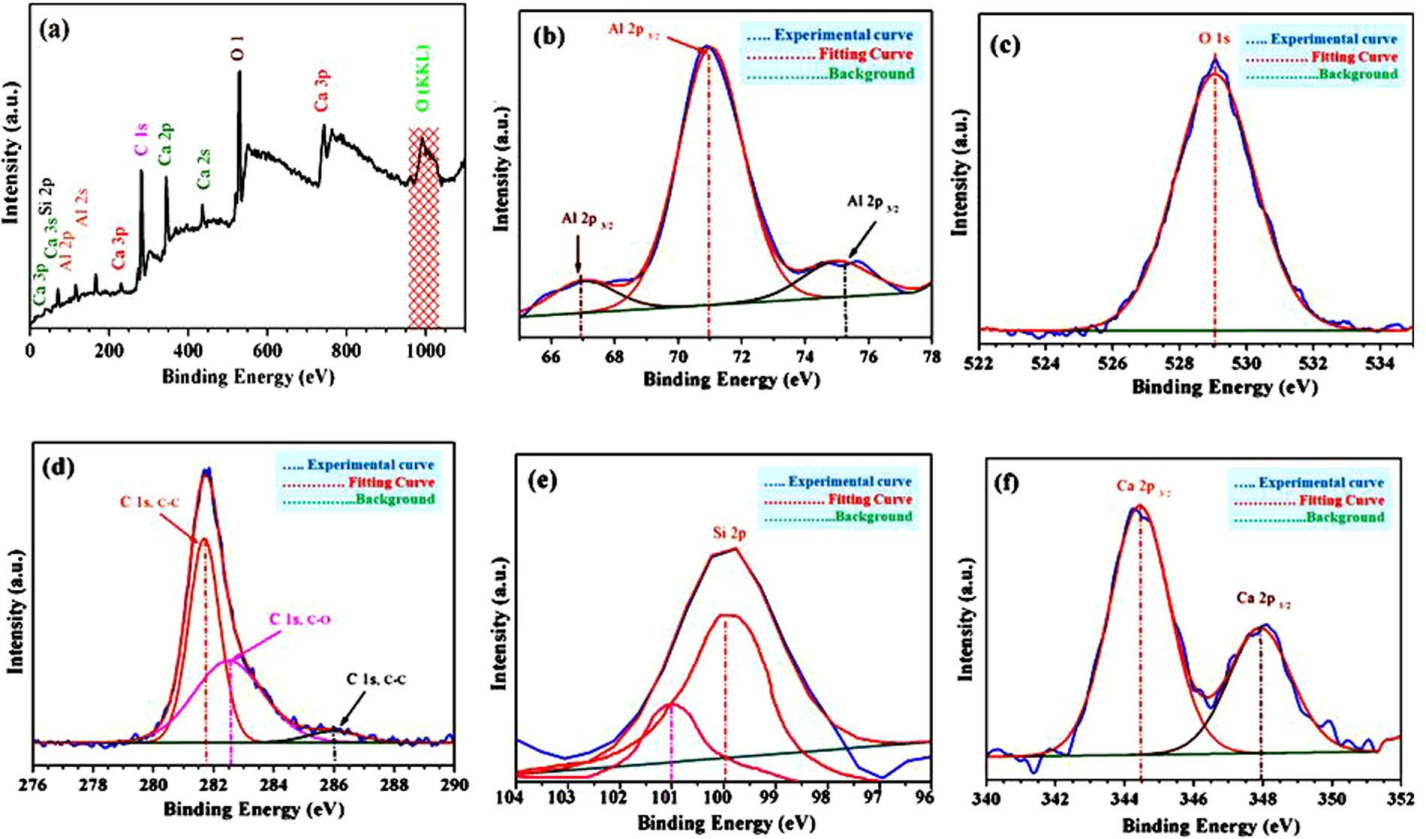
XPS spectra of Si-doped C12A7:e^−^ composite (x = 1), (**a**) full-scan, (**b**) Al 2p, (**c**) O 1 s, (**d**) C 1 s, (**e**) Si 2p, and (**f**) Ca 2p.

XPS technique was applied for elemental analysis where we apply Shirley background correction and Gaussian-Lorentzian peak shape for curve fitting of C 1 s. Figure 11(a) show XPS wide range scan data of Si-doped C12A7:e^−^ composite with in all required elements (Ca, Al, O, C, and Si). For further XPS based verification of the stoichiometric compositions of Si-doped C12A7:e^−^ based on binding energies related peaks positions of C 1 s, Ca 2p, Al 2p, O 1 s, and S1 2p were studied (Fig. 11(b–f)).

Based on those results, Al 2p peak was positioned at around 70.473 eV, shows that the valence state of Al in C12A7 is similar to that of Al_2_O_3_ (Fig. 11(b))^1,2^. In addition, the O 1 s spectrum the peak is at about 529.15 eV (Fig. 11(c))^36^. Also, the Ca 2p narrow XPS spectrum, along with profiles fits shows two peaks at around 344.57 eV and 348 eV, related to Ca 2p_1/2_ and Ca 2p_3/2_, respectively (Fig. 11(f)). Those are due to spin orbit splitting of the Ca 2p in XPS spectrum. Hence, these results shows that Ca is connected with O, making CaO^36^. For the rGO configuration and confirmation, we investigated the C 1 s peak data (Fig. 11(d)). The resulting peak after fitting was positioned at 281.95 eV (sp^2^, 47%)^37,38^. This peak was corresponds to non-oxygenated rings of carbon atoms in a conjugated honey comb lattice, shows that this highest peak ratio is due to formation of C-C skeleton by the reduction process of oxygen-containing species^39,40^. Similarly, oxygenated rings peaks at 282.59 eV, and 286.05 eV could be attributed to the C in C-O or C-OH (33%), and carbonyl (C = O, 20%), respectively^37,38^. The C/O ratios was ~13.5, which is almost similar to chemically produced rGO^41^. Hence these XPS results also support the Raman spectroscopy data for the formation of rGO on surface of Si-doped C12A7:e^−^ composite^37^ with some stable oxygenated functional groups remains preserved even after reduction^39^. Finally the positions of Si 2p along with other elements, Ca, Al, and O in XPS data, confirm the formation of Si-doped C12A7:e^−^ nano-particles with rGO coating on it, but this rGO were removed after melting of Si-doped C12A7:e^−^ nano-particles sample.

### Thin film deposition

In this part, we deposited thin film by magnetron sputtering method using Si-doped C12A7:e^−^ electride 3 inches target to explore its potential applications in optoelectronic devices. The sputtering process was conducted at 90 °C with the sputtering power set at 180 W for 10 mints.

**Figure 12:**
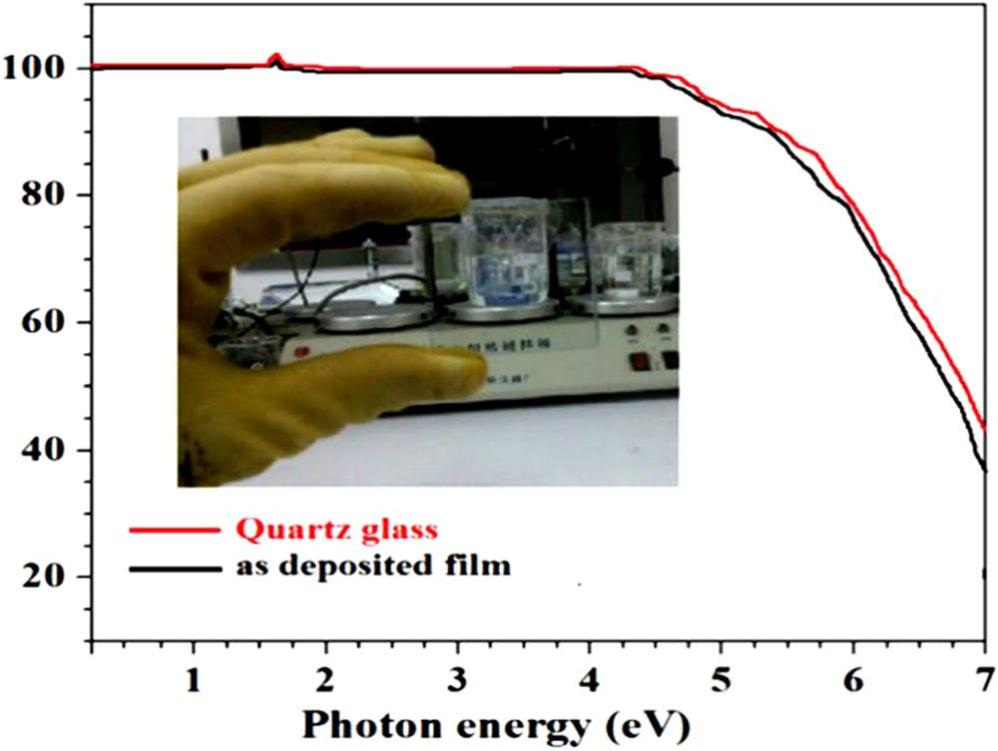
UV-Vis optical spectrum of as-deposited C12A7:e^−^ film, free of rGO.

First of all before we deposit the film we close the shutter and sputter the target for 10 mints to clean the surface of target and after that we started deposition by open the shutter. The UV-Vis optical transmittance + reflectance spectrum of as-deposited thin films with quartz substrate and measured thickness of grown thin film was about 390 nm and inset Fig. 12 shows optical photograph of the as-deposited thin film with highest achieved transparency. The energy band gap of electride thin film was calculated by Tauc’s formula, was about ~5.7 eV, which well fit with band gap value from cage conduction band to valence band, as previously reported^9^. For optoelectronic devices application, additional investigations are currently underway.

## Conclusion

In this paper we successfully synthesized doped-C12A7:e^−^ composites with different suitable elements and with/without rGO electride. In case of Sn-doped C12A7:e^−^ composite the conductivity was in the range of 1.6 S·cm^−1^ to 5.79 S·cm^−1^ but the melted rGO free sample with highest achieved conductivity of 280 S.cm^−1^ and the 3 inch size electride target has a relative density of 97 ± 0.5% was achieved. In case of the Si-doped C12A7:e^−^ composite the conductivity was in the range of 0.16 S·cm^−1^ to 1.75 S·cm^−1^, well agreed with previous results^32^ but melted sample with highest achieved conductivity of ~300 S.cm^−1^ and the 3 inch size electride target has a relative density of 99 ± 0.5%. Furthermore, transparent amorphous thin film was fabricated via magnetron sputtering, with almost zero percent losses. In conclusion, this optimized sol-gel method is suitable for doping different elements in C12A7:e^−^ to further alter the electrical and optical properties.
